# Road Environments: Impact of Metals on Human Health in Heavily Congested Cities of Poland

**DOI:** 10.3390/ijerph14070697

**Published:** 2017-06-29

**Authors:** Ewa Adamiec

**Affiliations:** Faculty of Geology, Geophysics and Environmental Protection, AGH University of Science and Technology, 30 Mickiewicza Av., 30-059 Kraków, Poland; eadamiec@agh.edu.pl

**Keywords:** oxidation stress, metals, non-carcinogenic health risk assessment, road dust, brake lining, tire, non-exhaust emission, road environment

## Abstract

Road dust as a by-product of exhaust and non-exhaust emissions can be a major cause of systemic oxidative stress and multiple disorders. Substantial amounts of road dust are repeatedly resuspended, in particular at traffic lights and junctions where more braking is involved, causing potential threat to pedestrians, especially children. In order to determine the degree of contamination in the heavily traffic-congested cities of Poland, a total of 148 samples of road dust (RD), sludge from storm drains (SL) and roadside soil (RS) were collected. Sixteen metals were analysed using inductively coupled plasma mass spectrometry (ICP-MS), inductively coupled plasma atomic emission spectroscopy (ICP-OES) and atomic absorption spectroscopy (AAS) in all samples. Chemical evaluation followed by Principal Component Analysis (PCA) revealed that road environments have been severely contaminated with traffic-related elements. Concentration of copper in all road-environment samples is even higher, exceeding even up to 15 times its average concentrations established for the surrounding soils. Non-carcinogenic health risk assessment revealed that the hazard index (HI) for children in all road-environment samples exceeds the safe level of 1. Therefore, greater attention should be paid to potential health risks caused by the ingestion of traffic-related particles during outdoor activities.

## 1. Introduction

Non-exhaust emission from traffic-related sources is considered the priority issue by the European Union within its sustainable transport strategy, since it greatly contributes to the overall problem of air pollution. However, despite the ongoing fight for better air quality, the vast majority of populations in urban areas is still exposed to air which does not comply with any World Health Organisation (WHO) Air Quality Guidelines and therefore causes a serious health threat. Kelly and Fussell [1] confirm that air pollution is a major cause of various health conditions such as pulmonary and systemic oxidative stress and inflammation, translocation of particle constituents and an associated risk of vascular dysfunction, atherosclerosis, altered cardiac autonomic function, ischaemic cardiovascular and obstructive pulmonary diseases, hemorrhagic stroke and increased cerebrovascular ischemia [2,3] or even cancer [4]. For the primary PM_10_, half of the airborne particles come from non-exhaust emissions originating from multiple sources, with the most significant one being brake lining wear. As reported by Garg et al. [5] and Kukutschová et al. [6], over 50% of brake debris becomes airborne, 80% of which is PM_10_ and 60% is PM_2.5_. According to Harrison et al. [7], in urban environments, brake wear can contribute up to 55% to the total non-exhaust traffic-related PM_10_ emissions and up to 21% to the total traffic-related PM_10_ emissions [7,8]. Garg et al. [5], Bukowiecki et al. [8] and Iijima et al. [9] estimate that about 50% of brake wear debris is deposited on roads or nearby. Road dust may also become contaminated in the process of tire and road-surface abrasion. According to Wikand Dave [10], followed by Panko et al. [11], 0.1–10% of tire debris becomes airborne. The rest is deposited on roads or in their close vicinity. Approximately 5% to 30% of non-exhaust traffic-related PM10 emissions can be attributed to tire wear particles [6], which are estimated to be 0.8% up to 7% by mass of ambient PM_10_. In terms of ambient concentrations, this corresponds to values ranging from 0.05 to 11 μg·m^−3^ [7,10,11,12]. Abrasion of tire tread generates coarse particles of 50–80 μm. These particles are composed mostly of minerals (61%), 13% carbon black, 16% polymers and 10% plasticisers and oils. The average mass of a new car tire is approximately 8 kg; during its lifetime it loses up to 1.5 kg. This means that within 3 years, 10–20% of the rubber enters the environment due to abrasion. It is estimated that about 90% of tire wear debris is deposited on the road or in close vicinity to roads. Very advanced studies on roadside pollution using test-plots were conducted by Wawer et al. [13]. Moreover, a substantial amount of road dust becomes resuspended and might be regarded as a secondary pollution source (50% according to Garg et al. [5]; Kukutschová et al. [6]; Bukowiecki et al. [8]).

According to Duong and Lee [14], the concentrations of heavy metals in road dust vary significantly depending on traffic and road features (roundabouts, motorway roads, traffic lights, etc.). Concentrations of metals in road dust from motorways are approximately twice the amount determined near roundabouts and downtown areas. During rapid braking, brakes are exposed to extensive heat from friction, which is transmitted to brake discs and results in the emission of particles. The most intense brake wear obviously occurs at intersections, corners, traffic lights, and through forced braking. According to Österle et al. [15], standard brake linings consist of 48% barite, 14% vermiculite, 19% phenolic resin, 4.6% antimonite, 5% rubber, 6.4% aramide, and 0.3% sulfur. The composition, function and friction testing of brake materials and additives have been discussed in detail by Chan and Stachowiak [16]. A wide variety of components are commonly used in vehicle brake lining, from steel or glass fibres and plastics that serve as reinforcements, to brass chips that are used for their heat-conducting properties. Authors such as Grigoratos and Martini [17] and Adamiec et al. [18] report that 90% of all metals originating from brake pads are bound to fraction < 20 μm. A wide variety of materials used in brakes contributes to the complexity of non-exhaust vehicle emissions. Most of them are a potential health hazard. Due to significant variability, identifying the chemical composition of road dust from vehicles is complex. Since most studies on toxicity and health consequences of roadside PM focus on exhaust emission, particles from the non-exhaust sources are yet to be better [19] and their health effects are disregarded in policy regulations despite obvious links with respiratory and cardiovascular health problems and, consequently, morbidity [20,21,22,23,24]. Airborne particles have been extensively examined by many researchers; however, particles from non-exhaust emissions, an important source of air pollution, have not been as well documented [7,25,26]. Extensive regulations on exhaust emission and technological improvements in the automobile industry have decreased the percentage of tailpipe emissions to total ambient PM concentrations [27,28,29]. The automotive industry has been forced to comply with environmental requirements since European Commission has established a set of Euro norms regarding LDVs (light duty vehicles) and HDVs (heavy duty vehicles). This has led to new developments in technologies employed to control exhaust emission, while non-exhaust particle emissions still remain high. Rexeis and Hausberger [30] estimated that by the end of this decade even 90% of the total emissions from road traffic may come from non-exhaust sources. With the current lack of any EU regulations, there is social interest in establishing a new set of recommendations regarding non-exhaust emissions. However, in order to provide general recommendations, it is necessary to broaden the existent knowledge by performing comprehensive studies on non-exhaust emission.

The main objective of the study was to evaluate metal contamination in various road-environment samples such as road dust, sludge from storm drains and roadside topsoil obtained from four of the biggest and highly congested cities in Poland, a country facing the worst air pollution problem in Europe. Werkenthin et al. [31] present a very interesting survey of advanced studies on metals in European roadside soils. Interestingly, there are fewer studies on road dust and only sporadic projects on sludge. However, in order to fully characterise roadside pollution, a complex analysis of sources other than soil (e.g., road dust or sludge from storm drains) is crucial. In the present research, concentrations of the selected metals Ba, Co, Cr, Mn, Ni, Se, Sn, Zr and well-known traffic indicators such as Cd, Cu, Pb, Sb, Ti, Zn were determined in all road environment samples. Data sets have been processed with the use of Principal Component Analysis (PCA). Chemical analyses have been supplemented by the calculation of pollution index (PI) for selected metals. Furthermore, the results were used to calculate an average daily dose through ingestion (ADD), hazards index (HI) and hazard quotient (HQ) for Ba, Cd, Co, Cu, Mn, Mo, Pb, Sb, Se, Sn, Zn and Zr and to assess the potential non-carcinogenic risk of traffic-related elements in road-environment samples, with adverse effects on child and adult health in particular.

## 2. Materials and Methods

### 2.1. Sampling Area

**Figure 1:**
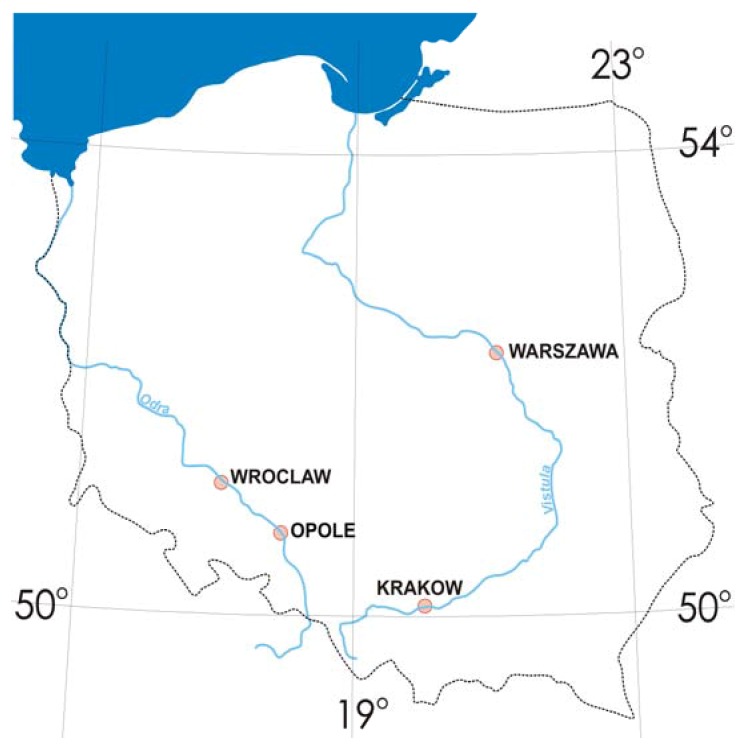
Sampling areas [32].

The most traffic-congested cities of Poland (Krakow, Opole, Wroclaw and Warszawa) were selected as research sites. Over the last two decades, all four have faced significant decline in heavy industry. In each city, eight sampling areas were selected near roads with very high road traffic, junctions, and heavy pedestrian traffic. They were located as far as possible from industrial plants and far from residential areas to minimise the impact of contamination sources. Samples were taken monthly starting from May to November 2015 to avoid the impact of other non-traffic-related pollutants, such as those emitted from household furnaces. Research areas are depicted on Figure 1 [32] and in Table 1. A total of 148 road-environment samples have been collected, including road sediment, sludge form storm drains and roadside topsoil (from 20 cm depth). The examined section of the road was straight, with no more than 3% slope, approx. 10–15 m in width with asphalt pavement surface and curbs of about 3–5 m width on each side of the road. Samples were taken after 5 days with no rainfall; temperatures varied between 8 °C and 24 °C. Samples of road dust (RD), including field duplicates, were collected in situ using a vacuum cleaner specifically modified for collecting road dust; some samples were sprayed with water and swept with a brush from the road (rectangle 4 m × 2 m). The results obtained with both methods were comparable. Sludge (SL) was sampled from storm drains in the amounts of about 1000 mg. Roadside topsoil (RS) was collected from square sections of 1 m × 1 m, at 20 cm depth (about 10 m away from the bridge).

### 2.2. Methods

Road dust, roadside topsoil and sludge from storm drains were digested with aqua regia according to 3050A protocol [33]. The concentrations for Ba, Cd, Co, Cr, Cu, Mn, Mo, Ni, Pb, Sb, Se, Sn, Ti, W, Zn, and Zr were then determined using inductively coupled plasma-mass spectrometry (ICP-MS) (ELAN 6100; Perkin Elmer, Waltham, MA, USA) according to U.S. EPA 6020B protocol [34]. The obtained data set has been processed with the use of PCA. PCA is one of the most common multivariate statistical methods used in environmental and geochemical studies [35,36,37,38]. All results were preprocessed and statistically evaluated according to van den Berg et al. [39] using Statistica 12.0 Software (STAT Lt., Ligonier, PA, USA). After centering and autoscaling the data, PCA was carried out to ascertain factors possibly contributing to concentrations of 11 metals in road dust, sludge from storm drains and roadside soil.

The concentrations of metals in road dust were then compared with median concentrations determined for the surrounding soils by Lis, Pasieczna [40] for Krakow, Tomassi-Morawiec et al. [41] for Warszawa, and Tomassi-Morawiec et al. [42] for Wroclaw and Opole.

Furthermore, PI (pollution index) was calculated from Equation (1):

PI = Ci/Ni(1)

where Ci is the concentration of metal and Ni stands for the geochemical background derived as the median concentration for the surrounding soils by Lis, Pasieczna [40] for Krakow, Tomassi-Morawiec et al. [41] for Warszawa, Tomassi-Morawiec et al. [42] for Wroclaw and Opole. PI index for each metal can be classified as non-pollution (PI ≤ 1), low level of pollution (1 < PI ≤ 2), moderate level of pollution (2 < PI ≤ 3), strong level of pollution (3 < PI ≤ 5) and very strong level of pollution (PI > 5) [43,44].

In order to assess the non-carcinogenic risk for children and adults from road dust, sludge from storm drains and roadside topsoil, an average daily intake dose of deleterious substances and exposure through ingestion (ADD) of Ba, Cd, Co, Cu, Mn, Mo, Ni, Pb, Sb, Se, Sn, Zn and Zr was calculated from the following formula according to U.S. EPA [45].

ADD = (Ci × IngR × EF × ED × CF)/(BW × AT)(2)

where Ci is the concentration of metal in the exposure site (mg/kg), (see Table 2); IngR is the ingestion rate 100 mg/day for children, and 50 mg/day for adults according to the recommended value for daily soil and dust ingestion [45,46,47]; EF is the exposure frequency of 350 days/year; BW is the average body weight 15 kg for children, 60 kg for adults; AT for non-carcinogenic (ED × 365 days); ED is the exposure duration; CF is the conversion factor 10^−6^ kg/mg. All parameters used in the calculation of ADD were found in U.S. EPA Exposure Handbook [45].

The potential non-carcinogenic risk of Ba, Cd, Co, Cu, Mn, Mo, Ni, Pb, Sb, Se, Sn, Zn and Zr was evaluated by the hazard quotient (HQ).

HQ = ADD/RfD(3)

where oral reference dose (RfD) was obtained from Regional Screening Levels (RSLs)-Generic Tables [48]. Since the RfD for Pb was not specified in RSLs U.S. EPA Report [49], in this study the RfD value is 3.5 × 10^−3^ mg/kg body weight per day according to [49,50,51,52,53,54].

If the HQ < 1, then non-carcinogenic toxic effects are unlikely. If HQ ≥ 1, then potential adverse health effects may occur. HQ > 10 suggests a high chronic risk [45]. Though interactions between metals might result in a synergic effect, the hazard index (HI) was used to assess the overall potential for non-carcinogenic effects of deleterious substances. HI, which is the sum of HQ was calculated from Equation (4):

(4) $$\mathrm{HI}=\overset{n}{\underset{i=1}{\sum }}\mathrm{HQi}$$

If HI < 1, then there is no significant risk. If HI > 1, there is a chance that non-carcinogenic toxic effects may be possible, with the increasing probability as HI increases [45,55].

### 2.3. Data Quality

In order to obtain unambiguous and unbiased ICP-MS results, elements were additionally measured using inductively coupled plasma-optical emission spectroscopy (ICP-OES) (OPTIMA 7300DV; Perkin Elmer, Waltham, MA, USA), atomic absorption spectroscopy (AAS) (F-AAS Thermo Scientific IC 3500, Waltham, MA, USA), according to U.S. EPA method 7000 in the Laboratory of Trace Analyses at the Faculty of Geology, Geophysics and Environmental Protection, AGH University of Science and Technology. Analyses were performed according to standard certified analytical quality control procedure (PN-EN ISO 17294-1:2007). Moreover, reagent blanks and certified international reference material METRANAL™ 32 (light sandy soil, grain size <100 µm; Analytika, Praha, Czech Republic) were used to ensure that the analytical results met the required criteria. Analyses of the reference material verified and confirmed the quality of the results. Analytical bias was statistically insignificant (*p* = 0.05) and the precisions of AAS and ICP-MS systems were satisfactory, which was verified by six different solution injections. Rh was used as an internal standard. Using ICP-MS, element correction equations were used for each element to minimise the impact of interferences.

## 3. Results and Discussion

### 3.1. Statistical Parameters of Traffic-Related Elements in Road Environments

Concentrations of Cr, Cu, Ni and Pb, Ti, Zn, Zr, well-known indicators of traffic pollution, were severely elevated in all types of road-environment samples, especially those collected in Warszawa, Krakow and Wroclaw. These are the biggest cities of Poland, which are densely populated and therefore greatly affected by traffic. Opole, on the other hand, where the concentrations of the investigated metals were much lower, is considered to be a medium-sized city with the population of 17 to 7 times smaller than Warszawa, Krakow or Wroclaw. As such it is less traffic-congested. Statistical parameters of the examined road-environment samples are presented in Table 2.

Table 3 presents metal contamination in road dust and topsoil samples collected in recreational areas in each of the four cities. They are considered to be unpolluted control samples. The top soil values are median concentrations obtained for the surrounding soils by Lis, Pasieczna [40] for Krakow; Tomassi-Morawiec et al. [41] for Warszawa; and Tomassi-Morawiec et al. [42] for Wroclaw and Opole.

### 3.2. Chemometric Analysis of Metal Concentrations in Road-Environment Samples

**Figure 2:**
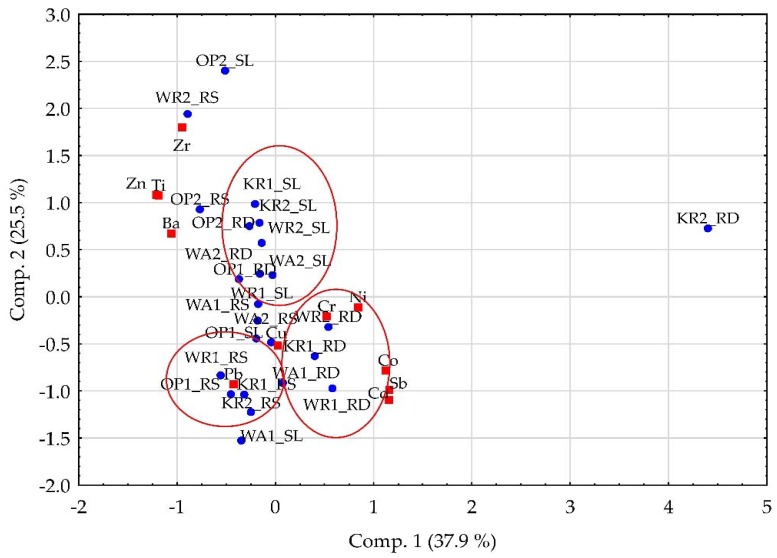
Principle component analysis (PCA) biplot for the first and second principle components (PCs) (scores and loadings).

PCA revealed two main components accounting for 63.36% of the total variance. Comp. 1 accounts for 37.84% of the total variance and is strongly positively correlated with Co, Cd, Sb and Ni. Furthermore, 25.52% of the variance is characterised by Comp. 2, which has a strong positive correlation with Ti, Zn and Zr. Score projection and loadings on the bi-dimensional space, defined by first two principal components, have been presented in Figure 2.

Samples can be grouped into three groups. On the right hand side of the bottom section in Figure 2, road dust (RD) samples (Positive Comp. 1, Negative Comp. 2) are grouped close to Cd, Co, Sb, Cu, Cr and Ni. Copper found in the road environment can primarily come from the wear of reinforcing fibres used in the form of chips or granules in brake linings as well as CuS used as a friction additive. Elevated concentrations of Cd in road-environment samples could be sourced from brake lining components such as plated brake parts, especially rotors in amounts as high as 39.4%, as reported by McKenzie et al. [55]. Cr in road-environment samples can originate from the erosion or abrasion of metal plating and bodywork, as well as Cr_2_O_3_ used as a frictional additive. Contamination of road dust with chromium can also be the result of Cr being one of the main components in alloys used to produce wrist pins and connecting rods. A portion of both Cr and Ni in road samples can come both from the sand paper effect of tires on yellow and red marking paint, and from abrasion of the grey paint and anticorrosive on guardrails. Moreover, Sb and Cu compounds such as CuS, Sb_2_S_3_ are commonly used as frictional additives in brake pads [16]. The second isolated group consists of sludge (SL) samples characterised by positive values for Comp. 1 and Comp. 2 of slightly negative values, which are located close to metals such as Zn, Ba, Zr and Ti. Contamination with Zn can be attributed to the wear and tear of tires, since ZnO and ZnS are added to activate vulcanisation in the tire tread; however, Zn could also come from traffic signals or guardrails, where it is found in easily mobilised forms affecting soil and water environments [56,57]. Elevated amounts of Ba in road dust could be a consequence of using BaSO_4_ to improve wear resistance, as well as from guardrails [33,57]. Severely elevated concentrations of Ti in all types of road-environment samples in the examined cities may be of anthropogenic origin [58]; they might be linked to the use of alkali metal titanates as inorganic fillers for the purpose of stabilizing friction coefficient. The third group consists of roadside soil samples strongly correlated with Pb. In road dust and sludge from drains, concentrations of Pb were significantly lower. This suggests that elevated concentrations of Pb in roadside soil may come from “historical” contamination, since this element was an important component of bearing alloys and was commonly used in wheel balancing weights (now replaced by zinc weights) or Pbantidetonant in the form of organic alkyllead [59] as a gasoline additive (this was officially banned in Poland in 2004). Detailed correlation analysis revealed that road dust from Nowohucki bridge significantly differed from other samples since it contained higher concentrations of Cd, Co, Mo, Ni, Pb, and Sb. During the sampling campaign, the nearby ArcelorMittal steel plant suffered a failure, which resulted in heavy emissions of dust (total of 3.7 tones) into the atmosphere [60]. The results obtained in the analysis could be related to this event.

### 3.3. Assessment of Road Environmental Pollution

The highest median concentrations of Zn were recorded in the sludge collected from Krakow (681 mg/kg) and Warszawa (650 mg/kg). Its concentrations in road dust from those two cities were considerably lower, 345 mg/kg and 276 mg/kg, respectively. Concentrations of copper in all road-environment samples were even higher, exceeding 8 to 19 times the average concentration established for the surrounding soils. In Krakow, the concentration of Cu ranges from 27.2 mg/kg in roadside soil up to 499 mg/kg in sludge, and in Warszawa it varies from 45.3 mg/kg in roadside soil up to 339 mg/kg in road dust. The highest concentrations of Cd in all road-environment samples were found in Krakow and Wroclaw. Similarly, the concentrations of Pb were the highest in sludge from storm drains and road dust collected in those two cities.

PI index calculated for road-environment samples in Warszawa was 9–13 for Cu, 3–7 for Cr, 1–4 for Pb, 7 for Ti, and, 3–10 for Zn. PI index determined for road-environment samples in Krakow was comparably high and equalled 10–15 for Cu, 3–9 for Cr, 3–4 for Pb, 10–20 for Ti and 3–6 for Zn. Additionally, high concentrations of Cd were also recorded in Krakow, especially in roadside soil (PI 5), indicating a high level of contamination. In Wroclaw, PI indicates an extremely high Cr and Ti contamination in all types of road-environment samples, ranging from 5–18 for Cr to 11–16 for Ti. PI indices for Cu and Zn are relatively high in Wroclaw, ranging between 2–4 for Cu and 4–6 for Zn. In Opole, PI indicates moderate to high contamination. Figure 3 shows pollution Index (PI) values for Ba, Cd, Co, Cr, Cu, Mn, Ni, Pb, and Ti in the examined road environment samples.

### 3.4. Non-Carcinogenic Health Risk Assessment

Potential non-carcinogenic health risk (HQ) and the hazard index (HI) for children in heavily congested road urban environments are presented in Table 4.

An average HQ index for children was calculated with respect to traffic-related elements in road dust samples collected in Krakow, Warszawa, Wroclaw, Opole; the results are presented in descending order: Zr (6.81 × 10^−1^) > Co (1 × 10^−1^) > Sb (1.32 × 10^−1^) > Se (8.33 × 10^−2^) > Pb (5.90 × 10^−2^) > Mo (5.57 × 10^−2^) > Zn (4.61 × 10^−2^) > Cu (1.23 × 10^−2^) > Cd (3.07 × 10^−3^) > Ba (2.77 × 10^−3^) > Sn (1.62 × 10^−4^). The results determined for sludge from drain storms were as follows: Zr (7.52 × 10^−1^) > Co (1.48 × 10^−1^) = Sb (1.48 × 10^−1^) > Se (1.21 × 10^−1^) > Zn (6.95 × 10^−2^) > Mo (6.83 × 10^−2^) > Pb (6.81 × 10^−2^) > Mn (5.58 × 10^−2^) > Cu (1.41 × 10^−2^) > Cd (5.42 × 10^−3^) > Ba (3.36 × 10^−3^) > Sn (2.05 × 10^−4^); for the roadside soil the results were the following: Zr (6.43 × 10^−1^) > (1.74 × 10^−1^) > Zn (1.52 × 10^−1^) > Co (1.25 × 10^−1^) > Sn (1.17 × 10^−1^) > Pb (9.89 × 10^−2^) > Se (8.34 × 10^−2^) > Mo (5.41 × 10^−2^) > Sb (5.07 × 10^−2^) > Cd (1.21 × 10^−2^) > Cu (7.20 × 10^−3^) > Ba (4.79 × 10^−3^). Table 5 presents non-carcinogenic HQ and the HI for adults calculated with respect to traffic-related elements in road environmental samples.

The results of calculations for road dust are presented in the descending order as follows: Zr (3.62 × 10^−1^) > Sb (8.17 × 10^−2^) > Co (6.95 × 10^−2^) > Se (4.54 × 10^−2^) > Mo (3.84 × 10^−2^) > Pb (3.44 × 10^−2^) > Mn (3.23 × 10^−2^) > Zn (2.54 × 10^−2^) > Mn (2.10 × 10^−2^) > Cu (7.42 × 10^−3^) > Cd (1.73 × 10^−3^) > Ba (1.73 × 10^−3^) > Sn (8.77 × 10^−5^). Results determined for samples of sludge from drain storm are as follows: Zr (1.08 × 10^−1^) > Co (9.52 × 10^−2^) > Sb (5.50 × 10^−2^) > Cu (3.78 × 10^−2^) > Se (1.49 × 10^−1^) > Mn (1.19 × 10^−1^) > Zn (9.63 × 10^−3^) > Pb (9.03 × 10^−3^) > Mo (2.79 × 10^−2^) > Cd (2.07 × 10^−2^) > Cu (1.41 × 10^−2^) > Cd (5.42 × 10^−3^) > Ba (3.36 × 10^−3^) > Sn (2.05 × 10^−4^); finally, for roadside soil the results were as follows: Zr (3.21 × 10^−1^) > Co (6.52 × 10^−2^) > Pb (5.98 × 10^−2^) > Se (4.68 × 10^−2^) > Mo (3.82 × 10^−2^) > Mn (2.92 × 10^−2^) Sb (2.79 × 10^−2^) > Zn (2.64 × 10^−2^) > Mn (1.82 × 10^−2^) > Cd (1.21 × 10^−2^) > Ba (3.73 × 10^−4^) > Sn (8.07 × 10^−5^).

HQ levels for traffic−related elements were found to be lower than 1.0 in all cases, which means that there was no non−carcinogenic toxic risks for any of the metals when analysed separately. This seemed true both for children and adults. HI index for adults determined for the selected metals (Ba, Cd, Co, Cu, Mn, Mo, Pb, Sb, Se, Sn, Zn and Zr) was also less than 1.0, therefore confirming that the exposure to environmental road samples was safe for adults. However, this was different for children, since HI index in that case exceeded the acceptable value of 1.0, signifying potential hazard. The HI values in road dust range from 1.08 (Krakow) up to 1.60 (Opole). In the sludge, they vary from 1.26 (Wroclaw) to 1.58 (Opole) and in roadside soil from 1.25 (Opole) to 1.83 (Krakow). Therefore, greater attention should be paid to adverse effects on children’s health caused by potential ingestion of traffic−related particles in road environments, e.g., at street crossings. It would also be important to determine the speciation of traffic−related metals in road environmental samples to better understand the health risks they pose for humans and the environment.

## 4. Conclusions

The results of chemical analysis, followed by the calculation of pollution index (PI) for various components of road environment in Warszawa, Krakow, Wroclaw and Opole revealed that road dust, mixed sludge and sediment from storm drains as well as roadside soils were significantly contaminated with all of the investigated elements, in particular with Cu, Pb, Ti and Zn. The highest concentrations were found in samples of sludge from drain storms collected from all investigated cities. Sludge samples were approximately 20% more contaminated with metals than road dust and roadside soils. PCA has revealed that both road dust and sludge are strongly correlated with elements derived mostly from brake pads, tires and as a result of road surface abrasion due to frictional effects.

HQ indices calculated for the analysed traffic−related elements were all lower than 1.0, potentially indicating non−carcinogenic effect. HI index for selected metals (Ba, Cd, Co, Cu, Mn, Mo, Pb, Sb, Se, Sn, Zn and Zr) for adults fell within the safe value. However, in the case of children, the HI values exceeded the safe level of 1.0 for road dust, sludge from storm drains and roadside topsoil in all investigated cities. Therefore, monitoring of road environments should be intensified, since road dust as a byproduct of exhaust and non−exhaust particulate emissions easily enters human airways and may have an adverse effect on health.

## Figures and Tables

**Figure 3:** Pollution Index (PI) in road environment.

**Table:** 

Element		Ba	Cd	Co	Cr	Cu	Mn	Ni	Pb	Ti	Zn
Krakow	Road dust	1	1	1	8	10	2	2	3	12	3
	Sludge	2	3	2	9	12	2	2	3	20	6
	Roadside soil	1	5	2	3	15	1	1	4	10	4
Warszawa	Road dust	2	0	2	7	11	13	1	1	7	4
	Sludge	2	1	2	7	13	18	1	2	7	10
	Road sidesoil	2	1	1	3	9	10	0	4	7	3
Wroclaw	Road dust	1	0	2	16	4	1	6	1	13	4
	Sludge	2	1	3	18	4	0	6	1	11	5
	Road sidesoil	3	3	2	5	2	1	2	2	16	6
Opole	Road dust	1	1	2	13	5	1	4	1	30	6
	Sludge	1	1	2	8	6	1	4	2	26	6
	Roadside soil	2	2	2	4	2	1	2	1	15	4
		PI ≤ 1	Low contaminated				3 < PI ≤ 5	Highly contaminated			
		1 < PI ≤ 3	Moderately contaminated				PI > 5	Extremely highly contaminated			

**Table 1:** Sampling point location.

City	Sampling Point Location (Bridge and Street Name)	Geographical Coordinates
Krakow	Most Debnicki (bridge on the Vistula River); Al. Mickiewicza (KR1)	50°03′196″N–19°55′726″E
	Most Nowohucki (bridge on the Vistula River); Nowohucka Street (KR2)	50°03′306″N–19°59′810″E
	Kopiec Kosciuszki (Relatively traffic unpolluted area)	50°03′245″N–19°53′467″E
Warszawa	Most Poniatowski (bridge on the Vistula River) (WA1)	52°14′097″N–21°02′301″E
	Most Gdanski (bridge on the Vistula River) (WA2)	52°15′589″N–21°00′456″E
	Fretta Street (Relatively traffic unpolluted area)	52°15′099″N–21°00′484″E
Opole	Niemodlinska Street and the bridge on the Odra River (OP1)	50°40′144″N–17°54′436″E
	Nysy Łuzyckiej Street and the bridge on the Odra River (OP2)	50°40′367″N–17°54′860″E
	Spacrowa Street (Relatively traffic unpolluted area)	50°39′433″N–17°55′354″E
Wroclaw	Most Warszawski (bridge on the Odra River) (WR1)	51°07′770″N–17°03′441″E
	Most Milenijny (bridge on the Odra River) (WR2)	51°08′027″N–16°59′578″E
	Bulwar Kulczynskiego(Relatively traffic unpolluted area)	51°06′920″N–17°02′304″E

**Table 2:** Statistical parameters of traffic-related elements in road environment.

Element (mg/kg)		Ba	Cd	Co	Cr	Cu	Mn	Mo	Ni	Pb	Sb	Se	Sn	Ti	W	Zn	Zr
**Krakow**																	
road dust (*n* = 11)	min	76.7	0.788	3.61	30.3	91.8	340	6.02	10.7	23	10.2	56.0	10.3	275	7.05	179	5.47
	max	142	2.89	13.1	215	397	1250	28.4	50.8	212	82.5	155	24.2	584	16.7	503	17.7
	median	114	1.15	6.92	97.7	167	667	8.19	21.6	89.5	21.5	104	16.4	511	10.0	345	10.8
sludge (*n* = 11)	min	162	2.22	7.66	78.8	149	884	3.21	28.2	78.4	12.8	129	16.6	732	15.0	587	13.9
	max	211	5.49	20.8	122	499	1652	65.3	35.3	311	32.2	251	26.3	937	22.4	749	19.5
	median	193	2.79	9.38	103	190	936	13.9	30.6	94.7	19.4	172	23.1	834	18.5	681	17.4
roadside soil (*n* = 11)	min	51.2	0.804	7.26	20.9	27.2	165	2.30	8.95	68.2	1.26	16.3	6.01	170	7.05	179	5.08
	max	208	11.2	20.7	75.1	181	597	19.4	26.4	606	8.66	170	14.4	561	19.8	756	12.0
	median	109	5.31	9.37	40.1	90.6	356	12.2	17.4	126	3.62	100	6.56	403	12.2	417	7.25
**Warszawa**																	
road dust (*n* = 11)	min	32.1	0.140	2.44	9.33	61.5	1182	131	2.13	13.1	7.87	7.96	14.5	277	3.40	131	6.20
	max	174	1.72	6.64	259	339	4090	329	20.1	143	26.2	171	32.2	585	18.2	683	13.8
	median	94.1	0.370	4.79	78.1	144	2986	256	6.96	26.1	15.4	73.1	24.0	499	7.55	276	9.85
sludge (*n* = 11)	min	83.3	0.430	3.40	53.5	106	3086	179	5.52	23.9	19.0	56.4	26.2	300	7.84	238	6.25
	max	206	2.59	9.55	189	285	5743	450	26.2	124	66.3	190	105	921	24.1	942	20.2
	median	113	0.940	6.67	72	173	4182	317	7.33	41	24.3	113	39.2	528	17.7	650	11.2
roadside soil (*n* = 11)	min	71.7	0.134	3.07	22.7	45.3	1363	211	2.71	23.1	1.95	51.6	11.9	379	5.82	125	7.10
	max	238	1.08	7.51	150	220	3898	403	32.3	141	12.8	175	22.4	577	24.6	1080	13.4
	median	117	0.438	3.25	27.5	118	2397	261	3.66	96.4	5.38	101	14.6	488	9.95	196	9.67
**Wroclaw**																	
road dust (*n* = 11)	min	52.1	0.070	3.35	29.3	17.2	117	2.37	8.92	11.7	1.34	24.5	7.39	215	2.77	71.7	6.61
	max	187	0.450	13.8	415	157	329	26.21	176	44.9	8.45	118	71.8	762	16.7	652	25.2
	median	108	0.350	7.54	140	49.4	236	9.39	58.2	22.0	3.65	77.8	19.7	484	7.38	201	9.58
sludge (*n* = 11)	min	97.1	0.174	6.37	40.8	37.9	245	3.10	30.2	32.9	4.85	25.5	10.6	550	5.38	191	7.07
	max	312	1.90	19.5	168	266	661	25.5	118	132	32.4	146	33.0	1402	28.2	1137	26.0
	median	171	0.813	12.1	85.0	120	422	8.04	63.7	69.9	17.2	87.4	18.2	916	15.0	567	14.8
roadside soil (*n* = 11)	min	134	0.900	5.17	17.9	11.9	330	1.32	9.52	30.5	1.34	22.0	7.3	472	6.15	135	6.52
	max	491	6.73	10.9	83.7	43.3	781	4.69	24.1	70.3	8.45	132	28	790	16.5	615	13.8
	median	266	2.60	8.50	45.0	26.4	519	2.56	18.4	48.6	3.65	78.5	14.4	605	9.43	311	10.7
**Opole**																	
road dust (*n* = 11)	min	99.7	0.210	10.3	63.2	45.4	353	4.06	35.4	18.7	3.60	n.d.	9.59	831	5.15	194	10.7
	max	171	1.26	19.0	937	276	685	97.5	596.8	98.7	16.1	198	42.3	1435	21.5	891	22.4
	median	114	0.630	11.2	113	73.05	406	6.45	45.0	37.9	7.14	76.4	16.7	1134	8.71	292	12.1
sludge (*n* = 11)	min	103	0.240	8.59	55.1	54.4	291	4.16	33.4	28.4	4.60	38.5	8.61	791	7.36	246	9.62
	max	147	2.05	32.7	96.7	279	505	23.4	65.0	586	14.8	176	44.2	1582	14.6	541	14.9
	median	129	0.766	10.6	73.0	85.5	432	5.91	42.4	45.5	7.11	131	19.9	959	9.93	304	10.9
roadside soil (*n* = 11)	min	141	0.560	5.71	19.3	10.4	241	1.68	13.2	17.9	0.734	5.54	6.30	461	5.06	146	6.44
	max	403	1.97	12.1	108	55.0	814	18.4	39.7	270	6.14	160	27.1	894	16.0	570	19.1
	median	198	1.37	7.42	37.5	28.7	445	2.87	19.6	34.7	4.30	61.8	12.7	570	7.89	231	9.8

n.d.—not detected.

**Table 3:** Metal contamination of relatively unpolluted control samples.

(mg/kg)	Ba	Cd	Co	Cr	Cu	Mn	Mo	Ni	Pb	Sb	Se	Sn	Ti	W	Zn	Zr
**Krakow**																
road dust (*n* = 2)	78.96	1.013	8.27	16.6	43.0	164	13.1	14.8	56.9	22.7	67.3	15.3	218	17.5	107	8.76
soil (*n* = 2)	70.54	0.76	6.18	19.6	33.7	385	2.53	14.9	65.5	2.64	78.8	7.10	116	9.94	85.6	8.22
top soil *	52.0	0.7	4.0	8.0	11.0	319	-	9.0	22.0	-	-	-	28	-	73	-
**Warszawa**																
road dust (*n* = 2)	76.2	1.0	14.0	43.3	43.2	214	6.14	25.2	86.5	12.4	73.3	21.4	954	42.9	105	9.3
soil (*n* = 2)	58.6	0.7	9.15	13.1	54.5	331	6.73	16.3	51.9	3.03	86.6	6.48	117	10.1	105	6.5
top soil **	39.0	0.50	2.00	7.0	9.0	157	-	5	15.00	-	-	-	50.0	-	44	-
**Wroclaw**																
road dust (*n* = 2)	69.0	0.083	5.56	60.79	27.2	280	5.43	34.3	13.0	2.76	56.8	21.2	201	4.99	87.7	6.88
soil (*n* = 2)	63.9	0.173	3.79	31.46	17.9	267	3.72	11.5	18.5	1.44	83.4	7.44	121	3.52	79.0	6.82
top soil ***	59	0.50	3.00	6	9	280	-	7	17	-	-	-	25.0	-	35	-
**Opole**																
road dust (*n* = 2)	112	0.812	9.85	57.6	69.4	289	7.08	25.4	59.3	18.3	77.0	14.2	705	20.7	99.4	10.4
soil (*n* = 2)	87.8	0.552	17.4	22.0	77.2	169	3.43	25.9	55.5	10.0	62.8	17.4	157	14.5	106.8	6.42
top soil ***	59	0.50	3.00	6	9	280	-	7	17	-	-	-	25.0	-	35	-

* median concentrations obtained for the surrounding soils by Lis, Pasieczna [40] for Krakow; ** median concentrations obtained for the surrounding soils by Tomassi-Morawiec et al. [41] for Warszawa; *** median concentrations obtained for the surrounding soils Tomassi-Morawiec et al. [42] for Wroclaw and Opole.

**Table 4:** Non-carcinogenic health risk (HQ) and the hazard index (HI) for children from metals in road environmental samples.

Elements			Ba	Cd	Co	Cu	Mn	Mo	Pb	Sb	Se	Sn	Zn	Zr	HI
Reference Dose (* RfD)			2.00 × 10^−1^	1.00 × 10^−3^	3.00 × 10^−4^	4.00 × 10^−2^	1.40 × 10^−1^	5.00 × 10^−3^	3.50 × 10^−3^	4.00 × 10^−4^	5.00 × 10^−3^	6.00 × 10^−1^	3.00 × 10^−2^	8.00 × 10^−5^	∑HQ_i_
Krakow	road dust	* ADD	4.37 × 10^−4^	4.41 × 10^−6^	2.65 × 10^−5^	6.41 × 10^−4^	2.56 × 10^−3^	3.14 × 10^−5^	3.43 × 10^−4^	8.25 × 10^−5^	3.99 × 10^−4^	6.29 × 10^−5^	1.32 × 10^−3^	4.14 × 10^−5^	
		HQI	2.19 × 10^−3^	4.41 × 10^−3^	8.85 × 10^−2^	1.60 × 10^−2^	1.83 × 10^−2^	6.28 × 10^−3^	9.81 × 10^−2^	2.06 × 10^−1^	7.98 × 10^−2^	1.05 × 10^−4^	4.41 × 10^−2^	5.18 × 10−^1^	1.08
	sludge	* ADD	7.40 × 10^−4^	1.07 × 10^−5^	3.61 × 10^−5^	7.29 × 10^−4^	3.59 × 10^−3^	5.33 × 10^−5^	3.63 × 10^−4^	7.44 × 10^−5^	6.60 × 10^−4^	8.86 × 10^−5^	2.61 × 10−3	6.67 × 10^−5^	
		HQI	3.70 × 10^−3^	1.07 × 10^−2^	1.20 × 10^−1^	1.82 × 10^−2^	2.56 × 10^−2^	1.07 × 10^−2^	1.04 × 10^−1^	1.86 × 10^−1^	1.32 × 10^−1^	1.48 × 10^−4^	8.71 × 10−2	8.34 × 10^−1^	1.53
	roadside soil	* ADD	4.18 × 10^−4^	2.03 × 10^−5^	3.61 × 10^−5^	3.48 × 10^−4^	1.37 × 10^−3^	4.68 × 10^−5^	4.83 × 10^−4^	1.38 × 10^−5^	3.84 × 10^−4^	2.53 × 10^−5^	1.60 × 10−3	2.80 × 10^−5^	
		HQI	2.09 × 10^−3^	2.03 × 10^−2^	1.20 × 10^−1^	8.69 × 10^−3^	9.75 × 10^−3^	9.36 × 10^−3^	1.38 × 10^−1^	3.45 × 10^−2^	7.67 × 10^−2^	4.69 × 10^−1^	4.69 × 10−1	4.69 × 10^−1^	1.83
Warszawa	road dust	* ADD	3.61 × 10^−4^	1.42 × 10^−6^	1.84 × 10^−5^	5.52 × 10^−4^	1.15 × 10^−2^	9.82 × 10^−4^	1.00 × 10^−4^	5.91 × 10^−5^	2.80 × 10^−4^	9.21 × 10^−5^	1.06 × 10^−3^	3.78 × 10^−5^	
		HQI	1.80 × 10^−3^	1.42 × 10^−3^	6.12 × 10^−2^	1.38 × 10^−2^	8.18 × 10^−2^	1.96 × 10^−1^	2.86 × 10^−2^	1.48 × 10^−1^	5.61 × 10^−2^	1.53 × 10^−4^	3.53 × 10^−2^	4.72 × 10^−1^	1.10
	sludge	* ADD	4.33 × 10^−4^	3.61 × 10^−6^	2.56 × 10^−5^	6.64 × 10^−4^	1.60 × 10^−2^	1.22 × 10^−3^	1.57 × 10^−4^	9.32 × 10^−5^	4.33 × 10^−4^	1.50 × 10^−4^	2.49 × 10^−3^	4.30 × 10^−5^	
		HQI	2.17 × 10^−3^	3.61 × 10^−3^	8.53 × 10^−2^	1.66 × 10^−2^	1.15 × 10^−1^	2.43 × 10^−1^	4.49 × 10^−2^	2.33 × 10^−1^	8.67 × 10^−2^	2.51 × 10^−4^	8.31 × 10^−2^	5.37 × 10^−1^	1.45
	roadside soil	* ADD	4.49 × 10^−4^	2.38 × 10^−6^	1.25 × 10^−5^	4.53 × 10^−4^	9.19 × 10^−3^	1.00 × 10^−3^	3.70 × 10^−4^	2.06 × 10^−5^	3.87 × 10^−4^	5.60 × 10^−5^	7.52 × 10^−4^	3.71 × 10^−5^	
		HQI	2.24 × 10^−3^	2.38 × 10^−3^	4.16 × 10^−2^	1.13 × 10^−2^	6.57 × 10^−2^	2.00 × 10^−1^	1.06 × 10^−1^	5.16 × 10^−2^	7.75 × 10^−2^	9.33 × 10^−5^	2.51 × 10^−2^	4.64 × 10^−1^	1.05
Wroclaw	road dust	* ADD	6.90 × 10^−4^	2.24 × 10^−6^	4.82 × 10^−5^	3.16 × 10^−4^	1.51 × 10^−3^	6.00 × 10^−5^	1.41 × 10^−4^	2.33 × 10^−5^	4.97 × 10^−4^	1.26 × 10^−4^	1.28 × 10^−3^	6.12 × 10^−5^	
		HQI	3.45 × 10^−3^	2.24 × 10^−3^	1.61 × 10^−1^	7.90 × 10^−3^	1.08 × 10^−2^	1.20 × 10^−2^	4.02 × 10^−2^	5.83 × 10^−2^	9.95 × 10^−2^	2.10 × 10^−4^	4.28 × 10^−2^	7.66 × 10^−1^	1.20
	sludge	* ADD	6.90 × 10^−4^	2.24 × 10^−6^	4.82 × 10^−5^	3.16 × 10^−4^	1.51 × 10^−3^	6.00 × 10^−5^	1.41 × 10^−4^	2.33 × 10^−5^	4.97 × 10^−4^	1.26 × 10^−4^	1.28 × 10^−3^	6.12 × 10^−5^	
		HQI	3.45 × 10^−3^	2.24 × 10^−3^	1.61 × 10^−1^	7.89 × 10^−3^	6.29 × 10^−2^	1.20 × 10^−2^	4.02 × 10^−2^	5.83 × 10^−2^	9.95 × 10^−2^	2.10 × 10^−4^	4.28 × 10^−2^	7.66 × 10^−1^	1.26
	roadside soil	* ADD	1.70 × 10^−3^	1.66 × 10^−5^	5.43 × 10^−5^	1.69 × 10^−4^	3.32 × 10^−3^	1.64 × 10^−5^	3.10 × 10^−4^	1.91 × 10^−5^	5.02 × 10^−4^	9.21 × 10^−5^	1.99 × 10^−3^	6.84 × 10^−5^	
		HQI	8.50 × 10^−3^	1.66 × 10^−2^	1.81 × 10^−1^	4.22 × 10^−3^	2.37 × 10^−2^	3.27 × 10^−3^	8.87 × 10^−2^	4.78 × 10^−2^	1.00 × 10^−1^	1.53 × 10^−4^	6.63 × 10^−2^	8.55 × 10^−1^	1.40
Opole	road dust	* ADD	7.29 × 10^−4^	4.22 × 10^−6^	7.16 × 10^−5^	4.67 × 10^−4^	2.60 × 10^−3^	4.12 × 10^−5^	2.42 × 10^−4^	4.56 × 10^−5^	4.88 × 10^−4^	1.07 × 10^−4^	1.87 × 10^−3^	7.74 × 10^−5^	
		HQI	3.64 × 10^−3^	4.22 × 10^−3^	2.39 × 10^−1^	1.17 × 10^−2^	1.85 × 10^−2^	8.25 × 10^−3^	6.92 × 10^−2^	1.14 × 10^−1^	9.77 × 10^−2^	1.78 × 10^−4^	6.22 × 10^−2^	9.67 × 10^−1^	1.60
	sludge	* ADD	8.25 × 10^−4^	5.11 × 10^−6^	6.78 × 10^−5^	5.47 × 10^−4^	2.76 × 10^−3^	3.77 × 10^−5^	2.91 × 10^−4^	4.54 × 10^−5^	8.37 × 10^−4^	1.27 × 10^−4^	1.94 × 10^−3^	6.97 × 10^−5^	
		HQI	4.12 × 10^−3^	5.11 × 10^−3^	2.26 × 10^−1^	1.37 × 10^−2^	1.97 × 10^−2^	7.54 × 10^−3^	8.31 × 10^−2^	1.13 × 10^−1^	1.67 × 10^−1^	2.12 × 10^−4^	6.48 × 10^−2^	8.71 × 10^−1^	1.58
	roadside soil	* ADD	1.27 × 10^−3^	8.95 × 10^−6^	4.73 × 10^−5^	1.83 × 10^−4^	2.84 × 10^−3^	1.85 × 10^−5^	2.22 × 10^−4^	2.75 × 10^−5^	3.95 × 10^−4^	8.12 × 10^−5^	1.48 × 10^−3^	6.26 × 10^−5^	
		HQI	6.33 × 10^−3^	8.95 × 10^−3^	1.58 × 10^−1^	4.59 × 10^−3^	2.03 × 10^−2^	3.71 × 10^−3^	6.34 × 10^−2^	6.87 × 10^−2^	7.90 × 10^−2^	1.35 × 10^−4^	4.92 × 10^−2^	7.83 × 10^−1^	1.25

* ADD (average daily dose through ingestion), * RfD (mg/kg body weight per day), HQI (unitless).

**Table 5:** Non-carcinogenic HQ and the HI for adults from metals in road environmental samples.

Elements			Ba	Cd	Co	Cu	Mn	Mo	Pb	Sb	Se	Sn	Zn	Zr	HI
* RfD			2.00 × 10^−1^	1.00 × 10^−3^	3.00 × 10^−4^	4.00 × 10^−2^	1.40 × 10^−1^	5.00 × 10^−3^	3.50 × 10^−3^	4.00 × 10^−4^	5.00 × 10^−3^	6.00 × 10^−1^	3.00 × 10^−2^	8.00 × 10^−5^	∑HQ_i_
Krakow	road dust	* ADD	7.81 × 10^−5^	7.88 × 10^−7^	4.74 × 10^−6^	4.57 × 10^−4^	5.61 × 10^−6^	1.48 × 10^−5^	6.13 × 10^−5^	1.47 × 10^−5^	7.12 × 10^−5^	1.12 × 10^−5^	2.36 × 10^−4^	7.40 × 10^−6^	
		HQI	3.90 × 10^−4^	7.88 × 10^−4^	1.58 × 10^−2^	1.90 × 10^−2^	1.12 × 10^−3^	1.34 × 10^−3^	1.75 × 10^−2^	3.68 × 10^−2^	1.42 × 10^−2^	1.87 × 10^−5^	7.88 × 10^−3^	9.25 × 10^−2^	2.07 × 10^−1^
	sludge	* ADD	1.32 × 10^−4^	1.92 × 10^−6^	6.44 × 10^−6^	6.41 × 10^−4^	9.52 × 10^−6^	2.10 × 10^−5^	6.49 × 10^−5^	1.33 × 10^−5^	1.18 × 10^−4^	1.58 × 10^−5^	4.66 × 10^−4^	1.19 × 10^−5^	
		HQI	6.61 × 10^−4^	1.92 × 10^−3^	2.15 × 10^−2^	2.67 × 10^−2^	1.90 × 10^−3^	1.91 × 10^−3^	1.85 × 10^−2^	3.32 × 10^−2^	2.36 × 10^−2^	2.64 × 10^−5^	1.55 × 10^−2^	1.49 × 10^−1^	2.94 × 10^−1^
	roadside soil	* ADD	7.47 × 10^−5^	3.63 × 10^−6^	6.44 × 10^−6^	2.44 × 10^−4^	8.36 × 10^−6^	1.19 × 10^−5^	8.63 × 10^−5^	2.47 × 10^−6^	6.85 × 10^−5^	4.52 × 10^−6^	2.86 × 10^−4^	5.00 × 10^−6^	
		HQI	3.73 × 10^−4^	3.63 × 10^−3^	2.15 × 10^−2^	1.02 × 10^−2^	1.67 × 10^−3^	1.08 × 10^−3^	2.47 × 10^−2^	6.16 × 10^−3^	1.37 × 10^−2^	7.53 × 10^−6^	9.52 × 10^−3^	6.25 × 10^−2^	1.55 × 10^−1^
Warszawa	road dust	* ADD	6.45 × 10^−5^	2.53 × 10^−7^	3.28 × 10^−6^	2.05 × 10^−3^	1.75 × 10^−4^	4.77 × 10^−6^	1.79 × 10^−5^	1.05 × 10^−5^	5.01 × 10^−5^	1.64 × 10^−5^	1.89 × 10^−4^	6.75 × 10^−6^	
		HQI	3.22 × 10^−4^	2.53 × 10^−4^	1.09 × 10^−2^	8.52 × 10^−2^	3.51 × 10^−2^	4.33 × 10^−4^	5.11 × 10^−3^	2.64 × 10^−2^	1.00 × 10^−2^	2.74 × 10^−5^	6.30 × 10^−3^	8.43 × 10^−2^	2.64 × 10^−1^
	sludge	* ADD	7.74 × 10^−5^	6.44 × 10^−7^	4.57 × 10^−6^	2.86 × 10^−3^	2.17 × 10^−4^	5.02 × 10^−6^	2.81 × 10^−5^	1.66 × 10^−5^	7.74 × 10^−5^	2.68 × 10^−5^	4.45 × 10^−4^	7.67 × 10^−6^	
		HQI	3.87 × 10^−4^	6.44 × 10^−4^	1.52 × 10^−2^	1.19 × 10^−1^	4.34 × 10^−2^	4.56 × 10^−4^	8.02 × 10^−3^	4.16 × 10^−2^	1.55 × 10^−2^	4.47 × 10^−5^	1.48 × 10^−2^	9.59 × 10^−2^	3.55 × 10^−1^
	roadside soil	* ADD	8.01 × 10^−5^	4.25 × 10^−7^	2.23 × 10^−6^	1.64 × 10^−3^	1.79 × 10^−4^	2.51 × 10^−6^	6.60 × 10^−5^	3.68 × 10^−6^	6.92 × 10^−5^	1.00 × 10^−5^	1.34 × 10^−4^	6.62 × 10^−6^	
		HQI	4.01 × 10^−4^	4.25 × 10^−4^	7.42 × 10^−3^	6.84 × 10^−2^	3.58 × 10^−2^	2.28 × 10^−4^	1.89 × 10^−2^	9.21 × 10^−3^	1.38 × 10^−2^	1.67 × 10^−5^	4.47 × 10^−3^	8.28 × 10^−2^	2.42 × 10^−1^
Wroclaw	road dust	* ADD	7.40 × 10^−5^	2.40 × 10^−7^	5.16 × 10^−6^	3.38 × 10^−5^	1.62 × 10^−4^	6.43 × 10^−6^	1.51 × 10^−5^	2.50 × 10^−6^	5.33 × 10^−5^	1.35 × 10^−5^	1.38 × 10^−4^	6.56 × 10^−6^	
		HQI	3.70 × 10^−4^	2.40 × 10^−4^	1.72 × 10^−2^	8.46 × 10^−4^	1.15 × 10^−3^	1.29 × 10^−3^	4.31 × 10^−3^	6.25 × 10^−3^	1.07 × 10^−2^	2.25 × 10^−5^	4.59 × 10^−3^	8.20 × 10^−2^	1.29 × 10^−1^
	sludge	* ADD	2.40 × 10^−7^	5.16 × 10^−6^	9.59 × 10^−5^	1.62 × 10^−4^	6.43 × 10^−6^	3.99 × 10^−5^	2.50 × 10^−6^	5.33 × 10^−5^	1.35 × 10^−5^	1.38 × 10^−4^	6.56 × 10^−6^	7.53 × 10^−6^	
		HQI	1.20 × 10^−6^	5.16 × 10^−3^	3.20 × 10^−1^	4.04 × 10^−3^	4.59 × 10^−5^	7.97 × 10^−3^	7.14 × 10^−4^	1.33 × 10^−1^	2.70 × 10^−3^	2.29 × 10^−4^	2.19 × 10^−4^	9.42 × 10^−2^	5.65 × 10^−1^
	roadside soil	* ADD	1.82 × 10^−4^	1.78 × 10^−6^	5.82 × 10^−6^	1.81 × 10^−5^	3.55 × 10^−4^	1.75 × 10^−6^	3.33 × 10^−5^	2.05 × 10^−6^	5.38 × 10^−5^	9.86 × 10^−6^	2.13 × 10^−4^	7.33 × 10^−6^	
		HQI	9.11 × 10^−4^	1.78 × 10^−3^	1.94 × 10^−2^	4.53 × 10^−4^	2.54 × 10^−3^	3.51 × 10^−4^	9.50 × 10^−3^	5.12 × 10^−3^	1.08 × 10^−2^	1.64 × 10^−5^	7.10 × 10^−3^	9.16 × 10^−2^	1.50 × 10^−1^
Opole	road dust	*ADD	7.81 × 10^−5^	4.52 × 10^−7^	7.67 × 10^−6^	5.00 × 10^−5^	2.78 × 10^−4^	4.42 × 10^−6^	2.60 × 10^−5^	4.89 × 10^−6^	5.23 × 10^−5^	1.14 × 10^−5^	2.00 × 10^−4^	8.29 × 10^−6^	
		HQI	3.90 × 10^−4^	4.52 × 10^−4^	2.56 × 10^−2^	1.25 × 10^−3^	1.99 × 10^−3^	8.84 × 10^−4^	7.42 × 10^−3^	1.22 × 10^−2^	1.05 × 10^−2^	1.91 × 10^−5^	6.67 × 10^−3^	1.04 × 10^−1^	1.71 × 10^−1^
	sludge	* ADD	8.84 × 10^−5^	5.48 × 10^−7^	7.26 × 10^−6^	5.86 × 10^−5^	2.96 × 10^−4^	4.04 × 10^−6^	3.12 × 10^−5^	4.86 × 10^−6^	8.97 × 10^−5^	1.36 × 10^−5^	2.08 × 10^−4^	7.47 × 10^−6^	
		HQI	4.42 × 10^−4^	5.48 × 10^−4^	2.42 × 10^−2^	1.46 × 10^−3^	2.11 × 10^−3^	8.08 × 10^−4^	8.90 × 10^−3^	1.22 × 10^−2^	1.79 × 10^−2^	2.27 × 10^−5^	6.94 × 10^−3^	9.33 × 10^−2^	1.69 × 10^−1^
	roadside soil	* ADD	1.36 × 10^−4^	9.59 × 10^−7^	5.07 × 10^−6^	1.97 × 10^−5^	3.05 × 10^−4^	1.99 × 10^−6^	2.38 × 10^−5^	2.95 × 10^−6^	4.23 × 10^−5^	8.70 × 10^−6^	1.58 × 10^−4^	6.71 × 10^−6^	
		HQI	6.78 × 10^−4^	9.59 × 10^−4^	1.69 × 10^−2^	4.91 × 10^−4^	2.18 × 10^−3^	3.97 × 10^−4^	6.79 × 10^−3^	7.36 × 10^−3^	8.47 × 10^−3^	1.45 × 10^−5^	5.27 × 10^−3^	8.39 × 10^−2^	1.33 × 10^−1^

* ADD, * RfD (mg/kg body weight per day), HQI (unitless).
